# Petermann's Dental Almanac for 1880

**Published:** 1880-04

**Authors:** 


*Petermann's Dental Almanac for* 1880. Frankfort, Germany. Another year's issue of this useful publication has been received. It contains an excellent portrait of the late Dr. Hermann Rottenstein, of Frankfort, who died August 13, 1879.

The annual publication of this small work is an honor to its author, and of great service to the profession in Europe. It is doing all that is possible against the sale of bogus diplomas prepared by an illegal institution in Philadelphia, and we trust that Dr. Petermann may not only succeed in his object, but that his efforts may be properly recognized by the regular members of the dental profession in Europe.

